# Females with hypermobile Ehlers–Danlos syndrome self-report more sexual problems than chronic pain controls without hypermobility, males, or patients with hypermobile spectrum disorders

**DOI:** 10.3389/frph.2026.1753684

**Published:** 2026-04-01

**Authors:** Cynthia E. Neville, Frances C. Wilson, DeLisa Fairweather, Melissa K. Caywood, Katherine Gegoutchadze, Lincoln E. Rozen, Nick A. Farahani, Chrisandra L. Shufelt, Dacre R. T. Knight, Shilpa N. Gajarawala, Katelyn A. Bruno

**Affiliations:** 1 Department of Physical Medicine and Rehabilitation, Mayo Clinic, Jacksonville, FL, United States; 2 Department of Cardiovascular Medicine, Mayo Clinic, Jacksonville, FL, United States; 3 Department of General Internal Medicine, Mayo Clinic, Jacksonville, FL, United States; 4 Center for Clinical and Translational Science, Mayo Clinic, Rochester, MN, United States; 5 Division of Cardiovascular Medicine, University of Florida, Gainesville, FL, United States

**Keywords:** hypermobile Ehlers–Danlos syndrome, hypermobility spectrum disorders, pain, sexual dysfunction, sexual health

## Abstract

**Background:**

Hypermobile Ehlers–Danlos syndrome (hEDS) and hypermobility spectrum disorders (HSDs) are heritable connective tissue disorders characterized by widespread fragile soft connective tissue affecting the skin, ligaments, joints, vasculature, and internal organs. Although hEDS and HSD are autosomal-dominant conditions and would be expected to display a 1:1 sex ratio, studies report higher prevalence in women.

**Objectives:**

The purpose of this exploratory study was to determine whether self-reported sexual problems differed between females and males diagnosed with hEDS or HSD compared with controls with chronic pain but no hypermobility.

**Methods:**

In this exploratory retrospective study, we examined 1,407 patients diagnosed with hEDS or HSD according to the 2017 diagnostic criteria for eight indicators of sexual problems based on a validated screening tool.

**Results:**

Patients in each diagnosis primarily self-reported as White, non-Hispanic females (90%). Of the 1,407 patients who attended the EDS Clinic, 976 (69%) were diagnosed with HSD, 240 (17%) with hEDS, and 191 (14%) were chronic pain controls with neither diagnosis. For HSD, 937 (96%) were females vs. 39 (4%) males (24:1 females to males), while for hEDS, 210 (88%) were females vs. 30 (13%) males (7:1 females to males), and controls were 165 (86%) females vs. 26 (14%) males (6:1 females to males). Females with hEDS reported significantly higher rates of sexual issues than chronic pain controls, including sexual problems (69%, *p* = 0.018), problems with sexual interest (41%, *p* = 0.023), sexual pain (45%, *p* = 0.006), and orgasm difficulty (34%, *p* = 0.022) compared with males with hEDS or males or females with HSD. In contrast, males diagnosed with hEDS did not report any sexual problems over controls, and males and females diagnosed with HSD reported only one issue—more problems with sexual interest—compared with controls (females *p* = 0.019, males *p* = 0.018). However, the small number of males in this study limits conclusions that can be drawn for them.

**Conclusions:**

This study is unique because we examine sex differences in sexual problems in patients with hEDS and HSD. Our findings indicate a higher percentage of sexual problems in females with hEDS than chronic pain controls without hypermobility, but this was not observed for females with HSD.

## 1 Introduction

Ehlers–Danlos syndromes (EDS) comprise a heterogeneous group of mostly rare heritable genetic connective tissue/collagen disorders characterized by fragile soft connective tissue and widespread distribution that manifests in the skin, ligaments, joints, blood vessels, and internal organs (1). In 2017, new diagnostic criteria for hypermobile EDS (hEDS) were devised that distinguish hEDS from hypermobility spectrum disorders (HSDs) with a goal of better understanding the similarities and/or differences that exist within the spectrum of disease (2, 3). The criteria for a diagnosis of hEDS briefly include identification of generalized joint hypermobility (GJH) of specific joints using the Beighton scale, evidence of a systemic connective tissue disorder, family history and/or musculoskeletal complications, and several exclusions (3). Patients are diagnosed with HSD if they do not meet the diagnostic criteria for hEDS, have a positive Beighton score, and also have evidence that the joint hypermobility is causing widespread symptoms and is not just an asymptomatic feature (2–4). hEDS and HSD are estimated to effect 3% or 255 million people worldwide (2, 5, 6). However, this estimate is based on survey data from the UK, and prevalence data for hEDS and HSD are not available for most countries in the world. Although the genetic variants responsible for most types of EDS have been identified, the variant/s responsible for hEDS is unknown (7). As autosomal-dominant genetic conditions, hEDS and HSD would be expected to display a 1:1 sex ratio, but numerous studies have revealed a sex ratio of at least 9:1 female to male (8–10). Comorbidities associated with hEDS/HSD include subluxations, dislocations, chronic pain, fatigue, headache, migraine, mast cell activation syndrome (MCAS), anxiety, neurological and gastrointestinal symptoms, and other issues leading to a reduced quality of life (7, 10–15). Because of the complexity of hEDS/HSD and few specialists to treat these conditions, survey data indicate that it can take between 14 and 28 years to obtain a diagnosis (6).

Healthy connective tissue/collagen is essential to pelvic and genitourinary function and health. A number of studies describe pelvic and genitourinary dysfunction in patients with connective tissue disorders such as hEDS and HSD (16–18). Many reports describe greater pelvic girdle pain during pregnancy in individuals with GJH. A survey by Ahlqvist et al. of 2,445 women who were given the 5-point GJH questionnaire to determine hypermobility found that more women with GJH self-reported pelvic girdle pain during pregnancy (47%) than controls (41%) (19, 20). A large survey of 947 women with hEDS/HSD found an increased risk of maternal and neonatal complications compared with the general population (21). A qualitative study of 40 patients with hEDS/HSD found that the majority of participants recounted a worsening of symptoms during pregnancy and postnatal complications (22). Survey data from approximately 1,300 women who self-reported a diagnosis of hEDS revealed pelvic floor dysfunction that was higher than the general population, including stress urinary incontinence (60%), urgency urinary incontinence (54%), sexual dysfunction (49%), fecal incontinence (24%), and pelvic organ prolapse (21%) (23). A 2025 report of an online survey found that women who self-reported a diagnosis of HSD or hEDS (*n* = 84) had decreased scores on the Female Sexual Function Index (FSFI) in the subdomains of desire, arousal, lubrication, orgasm, and sexual satisfaction compared with self-reported healthy controls (*n* = 75) (24). Another study found that vulvodynia (debilitating vulvar pain accompanied by dyspareunia or pain with sexual intercourse) was reported to occur more often in patients who self-reported a diagnosis of hEDS/HSD (50%) (*n* = 1,146) than the general population (8%) (25). To date, the impact of pelvic floor symptoms and/or sexual problems has not been reported in males with HSD or hEDS. Information on urogenital and sexual problems in HSD and hEDS is limited, is dominated by observational studies and case reports, and studies do not differentiate between HSD and hEDS (17). The purpose of this exploratory study was to determine whether self-reported sexual problems differed between females and males diagnosed with hEDS or HSD compared with controls with chronic pain but no hypermobility.

## 2 Materials and methods

### 2.1 Ethics approval and consent to participate

The Institutional Review Board (IRB# 19-011260) of Mayo Clinic approved the retrospective analysis of demographic and clinical data from medical records for this study and waived informed consent for all patients. The research conformed to the principles outlined in the Declaration of Helsinki.

### 2.2 Study design

This retrospective observational study examined adult patients (≥18 years of age) seen at the Mayo Clinic Florida EDS Clinic in the Department of Internal Medicine from 1 February 2020 to 19 May 2025 (*n* = 1,407) by self-referral or referrals from inside or outside the Mayo Clinic. Patients were diagnosed with hEDS or HSD according to the 2017 diagnostic criteria (3, 4, 26) by EDS physician experts as previously (10, 27). Briefly, the diagnostic criteria for hEDS include identification of GJH of specific joints using the Beighton Scale (past puberty ≥5/9 and over 50 years of age ≥4/9), evidence of a systemic connective tissue disorder, family history and/or musculoskeletal complications, and several exclusions (3). HSD is diagnosed in patients who do not meet the diagnostic criteria for hEDS but have a positive Beighton score (≥5/9 and ≥4/9 for older patients) and evidence that the joint hypermobility is causing widespread problems and is not just an asymptomatic feature (feature C of the second EDS criterion) (3, 4, 26). Localized HSD (L-HSD) is diagnosed in patients who do not meet the diagnostic criteria for hEDS, do not have a positive Beighton score (but their Beighton score is not zero), and other areas of the body are hypermobile (4, 26, 27). Historical HSD (H-HSD) is diagnosed in patients who do not meet the diagnostic criteria for hEDS, have a Beighton score of zero, and other areas of the body are hypermobile (positive for the 5-point GJH questionnaire) (4, 19, 26). We did not include L-HSD or H-HSD in this assessment.

### 2.3 Controls

Controls in this study were patients seen at the EDS Clinic by our EDS physician experts but who did not receive a diagnosis of hEDS, HSD, L-HSD, or H-HSD or any other forms of EDS as previously (10, 27). Non-hypermobile controls were patients who generally reported widespread pain and were not healthy, but without a clear diagnosis. The advantage of using these patients as controls is that they received the same diagnostic assessment as patients with hEDS and HSD, so that we can be certain that they are not hypermobile. They were also given the same Intake Questionnaire given to patients with hEDS and HSD. In addition, they may be considered more ideal controls than healthy controls who are likely to have no sexual issues.

### 2.4 Patients with sexual problems

Adult patients (≥18 years of age) were seen at the Mayo Clinic Florida EDS Clinic from 1 February 2020 to 19 May 2025 (*n* = 1,407) and diagnosed with HSD or hEDS according to the 2017 diagnostic criteria as previously (3, 4, 26). As part of the EDS Clinic Intake Questionnaire (10, 13, 27), patients received a question about whether they had been sexually active in the past 30 days. In addition, patients received questions that screened for sexual problems: “In the past 12 months, has there ever been a period of 3 months or more when you had any of the following problems or concerns (sexual problems) (check all that apply): you wanted to feel more interest in sexual activity (sexual interest problems), you had pain during or after sexual activity (sexual pain), you had difficulty having an orgasm (orgasm difficulty), you felt anxious about sexual activity (sexual anxiety), you did not enjoy sexual activity (sexual pleasure), your vagina felt too dry (vaginal dryness) (women only), you had difficulty with erections (erection difficulty) (men only), some other sexual problem or concern (other), no sexual problems or concerns”. These questions were obtained from Table 6 of Flynn et al. who developed and validated a self-reported screening tool for sexual problems (28).

### 2.5 Genitourinary patients

Adult patients (≥18 years of age) were seen at the Mayo Clinic Florida EDS Clinic from November 1, 2019, to April 25, 2025 (*n* = 1,216) and diagnosed with HSD or hEDS according to the 2017 diagnostic criteria as previously (3, 4, 26). As part of the Intake Questionnaire, patients were asked whether they had any of the following genitourinary issues: frequent urination, dyspareunia (pain during sexual intercourse), recurrent urinary infections, incontinence (urine leakage), pelvic floor dysfunction, recurrent yeast infections, recurrent vaginal bacterial infections, pelvic floor spasms, interstitial cystitis (inflammation of the bladder), bladder prolapse, uterine prolapse, rectal prolapse, other genitourinary issues, unknown issues, and no genitourinary issues.

### 2.6 Data collection

Patients who attended the Mayo Clinic Florida EDS Clinic received a REDCap Intake Questionnaire as standard of care prior to their first appointment at the Mayo Clinic Florida EDS Clinic as previously (10, 13, 27). Adult patients who answered questions related to sexual activity, sexual problems, and/or genitourinary problems were included in the study.

### 2.7 Statistical analysis

Because this study is an exploratory evaluation of sexual problems in female and male patients with hEDS and HSD, we did not perform multiple comparison tests related to multiple symptoms because it may lead to type II errors (false negatives). Continuous variables (i.e., age) were summarized with the sample median and range. Categorical variables were summarized with the number and percentage of subjects. *p*-values for continuous variables were calculated using a Student's t-test for parametric data and a Mann–Whitney test for non-parametric data. A Fisher's exact test was used to determine *p*-values for categorical variables. *p*-values <0.05 were considered statistically significant. All statistical analyses were performed using GraphPad PRISM, version 10.6.0.

## 3 Results

### 3.1 Demographics

Of the 1,407 patients in this study who attended the Mayo EDS Clinic, 976 (69.4%) were diagnosed with HSD, 240 (17.1%) with hEDS, and 191 (13.6%) were chronic pain controls with neither diagnosis. For HSD, 937 (96.0%) were females vs. 39 (4.0%) males (24:1 female to male), while for hEDS, 210 (87.5%) were females vs. 30 (12.5%) males (7:1 female to male), and chronic pain controls with neither diagnosis were 165 (86.4%) females vs. 26 (13.6%) males (6:1 female to male).

We found that females with HSD (35.3, 18.0–70.9) or hEDS (35.6, 18.2–71.3) were younger than chronic pain controls (40.6, 18.0–76.5) (*p* < 0.001) (Supplementary Table S1). Overall, approximately 90% of females were White, non-Hispanic. More females with hEDS reported being past smokers (20.0%) vs. controls (11.5%) (*p* = 0.034) and currently consuming alcohol (61.9%) vs. controls (52.7%) (*p* = 0.002) (Supplementary Table S1). There were no significant differences in the highest level of education or secondhand smoke exposure history. Similarly, we found that males with HSD (33.3, 18.5–81.9) or hEDS (28.1, 18.0–52.0) were younger in age than controls (37.9, 18.6–68.6) (*p* = 0.018), with patients with hEDS significantly younger than controls (*p* = 0.014) (Supplementary Table S2). Like females, males were 87%–97% White, non-Hispanic. There were no significant differences in any of the other demographic parameters for males.

### 3.2 Sexual activity

In this study, we found that approximately 50% of female chronic pain controls and patients with HSD and hEDS reported being sexually active (Tables 1, 2). There was no significant difference between controls and females with hEDS/HSD in sexual activity. In contrast, approximately 60% of chronic pain control males and 40% of males with HSD or hEDS reported being sexually active. Although differences existed between males with hEDS/HSD and controls in sexual activity, they were not significantly different (Tables 1, 2). There were also no significant differences in sexual activity between females with hEDS and females with HSD (*p* = 0.76) or males with hEDS and males with HSD (*p* = 0.81) (Table 3) or sex differences in sexual activity by diagnosis: HSD female vs. male (*p* = 0.14); hEDS female vs. male (*p* = 0.12) (Table 4).

**Table 1:** Chronic pain controls vs. HSD.

Sexual symptoms	Female control^a^ (*n* = 165)	Female HSD^a^ (*n* = 937)	Female *p*-value^b^	Male control^a^ (*n* = 26)	Male HSD^a^ (*n* = 39)	Male *p*-value^b^
Sexual activity	82 (49.7)	531 (56.7)	0.11	16 (61.5)	17 (43.6)	0.21
Sexual problems	94 (57.0)	591 (63.1)	0.14	10 (38.5)	21 (53.9)	0.31
Sexual interest problems	49 (29.7)	370 (39.5)	**0.019** ^c^	2 (7.7)	13 (33.3)	**0.018**
Sexual pain	51 (30.9)	356 (38.0)	0.10	6 (23.1)	12 (30.8)	0.58
Orgasm difficulty	38 (23.0)	259 (27.6)	0.25	3 (11.5)	7 (18.0)	0.73
Sexual anxiety	27 (16.4)	219 (23.4)	0.05	3 (11.5)	8 (20.5)	0.50
Sexual pleasure	27 (16.4)	172 (18.4)	0.59	1 (3.9)	5 (12.8)	0.39
Other	7 (4.2)	65 (6.9)	0.23	3 (11.5)	4 (10.3)	0.99
Vaginal dryness	50 (30.3)	281 (30.0)	0.93	—	—	n/a
Erection difficulty	—	—	n/a	7 (26.9)	8 (20.5)	0.56

^a^ Data presented as *n* (%).

^b^ *p*-values obtained using Fischer's exact test.

^c^ Bold indicates significant value.

**Table 2:** Chronic pain controls vs. hEDS.

Sexual symptoms	Female control^a^ (*n* = 165)	Female hEDS^a^ (*n* = 210)	Female *p*-value^b^	Male control^a^ (*n* = 26)	Male hEDS^a^ (*n* = 30)	Male *p*-value^b^
Sexual activity	82 (49.7)	116 (55.2)	0.30	16 (61.5)	12 (40.0)	0.18
Sexual problems	94 (57.0)	145 (69.1)	**0.018** ^c^	10 (38.5)	13 (43.3)	0.79
Sexual interest problems	49 (29.7)	87 (41.4)	**0.023**	2 (7.7)	8 (26.7)	0.09
Sexual pain	51 (30.9)	95 (45.2)	**0.006**	6 (23.1)	5 (16.7)	0.74
Orgasm difficulty	38 (23.0)	72 (34.3)	**0.022**	3 (11.5)	3 (10.0)	0.99
Sexual anxiety	27 (16.4)	52 (24.8)	0.06	3 (11.5)	3 (10.0)	0.99
Sexual pleasure	27 (16.4)	49 (23.3)	0.12	1 (3.9)	2 (6.7)	0.99
Other	7 (4.2)	17 (8.1)	0.14	3 (11.5)	0 (0.0)	0.09
Vaginal dryness	50 (30.3)	62 (29.5)	0.91	—	—	n/a
Erection difficulty	—	—	n/a	7 (26.9)	30 (20.0)	0.75

^a^ Data presented as *n* (%).

^b^ *p*-values obtained using Fischer's exact test.

^c^ Bold indicates significant value.

**Table 3:** HSD vs. hEDS.

Sexual symptoms	Female HSD^a^ (*n* = 937)	Female hEDS^a^ (*n* = 210)	Female *p*-value^b^	Male HSD^a^ (*n* = 39)	Male hEDS^a^ (*n* = 30)	Male *p*-value^b^
Sexual activity	531 (56.7)	116 (55.2)	0.76	17 (43.6)	12 (40.0)	0.81
Sexual problems	591 (63.1)	145 (69.1)	0.11	21 (53.9)	13 (43.3)	0.47
Sexual interest problems	370 (39.5)	87 (41.4)	0.64	13 (33.3)	8 (26.7)	0.61
Sexual pain	356 (38.0)	95 (45.2)	0.06	12 (30.8)	5 (16.7)	0.26
Orgasm difficulty	259 (27.6)	72 (34.3)	0.06	7 (18.0)	3 (10.0)	0.50
Sexual anxiety	219 (23.4)	52 (24.8)	0.65	8 (20.5)	3 (10.0)	0.37
Sexual pleasure	172 (18.4)	49 (23.3)	0.10	5 (12.8)	2 (6.7)	0.69
Other	65 (6.9)	17 (8.1)	0.55	4 (10.3)	0 (0.0)	0.13
Vaginal dryness	281 (30.0)	62 (29.5)	0.93	—	—	n/a
Erection difficulty	—	—	n/a	8 (20.5)	30 (20.0)	0.99

^a^ Data presented as *n* (%).

^b^ *p*-values obtained using Fischer's exact test.

**Table 4:** Sex differences in patients with HSD and hEDS.

Sexual symptoms	Female HSD^a^ (*n* = 937)	Male HSD^a^ (*n* = 39)	Female *p*-value^b^	Female hEDS^a^ (*n* = 210)	Male hEDS^a^ (*n* = 30)	Male *p*-value^b^
Sexual activity	531 (56.7)	17 (43.6)	0.14	116 (55.2)	12 (40.0)	0.12
Sexual problems	591 (63.1)	21 (53.9)	0.24	145 (69.1)	13 (43.3)	**0.007** ^c^
Sexual interest problems	370 (39.5)	13 (33.3)	0.51	87 (41.4)	8 (26.7)	0.16
Sexual pain	356 (38.0)	12 (30.8)	0.40	95 (45.2)	5 (16.7)	**0.003**
Orgasm difficulty	259 (27.6)	7 (18.0)	0.20	72 (34.3)	3 (10.0)	**0.006**
Sexual anxiety	219 (23.4)	8 (20.5)	0.85	52 (24.8)	3 (10.0)	0.10
Sexual pleasure	172 (18.4)	5 (12.8)	0.52	49 (23.3)	2 (6.7)	0.05
Other	65 (6.9)	4 (10.3)	0.35	17 (8.1)	0 (0.0)	0.14
Vaginal dryness	281 (30.0)	—	n/a	62 (29.5)	—	n/a
Erection difficulty	—	8 (20.5)	n/a	—	30 (20.0)	n/a

^a^ Data presented as *n* (%).

^b^ *p*-values obtained using Fischer's exact test.

^c^ Bold indicates significant value.

### 3.3 Sexual problems in patients with HSD or hEDS vs. chronic pain controls by sex

We asked eight validated screening questions for sexual problems that included a female-specific symptom of vaginal dryness and a male-specific symptom of erection difficulty (Tables 1, 2). The only significant difference observed in patients with HSD was problems with sexual interest, which was significantly increased for both sexes compared with chronic pain controls (HSD females vs. controls odds ratio (OR) 1.55, confidence interval (CI) 1.09–2.21, *p* = 0.019; HSD males vs. controls OR 6.00, CI 1.21–28.29, *p* = 0.018) (Table 1, Supplementary Table S3). In contrast, females with hEDS reported many sexual problems, while males with hEDS reported no issues compared with chronic pain controls (Table 2, Supplementary Table S3). Females with hEDS reported more sexual problems (hEDS vs. controls OR 1.69, CI 1.11–2.58, *p* = 0.018), problems with sexual interest (hEDS vs. controls OR 1.67, CI 1.09–2.56, *p* = 0.023), sexual pain (hEDS vs. controls OR 1.85, CI 1.21–2.80, *p* = 0.006), and orgasm difficulty (hEDS vs. controls OR 1.74, CI 1.11–2.77, *p* = 0.022) vs. chronic pain controls (Table 2, Supplementary Table S3). Thus, females with hEDS reported more sexual problems than chronic pain controls with no hypermobility.

### 3.4 Sexual problems in patients with HSD and hEDS by sex

When we compared females with HSD and females with hEDS and males with HSD and males with hEDS, we did not find a significant difference between the two diagnoses for any of the sexual problem screening questions (Table 3). However, when we compared females with HSD and males with HSD and females with hEDS and males with hEDS, we found that females with hEDS reported more sexual issues than males, including sexual problems (hEDS females vs. males OR 2.92, CI 1.33–6.25, *p* = 0.007), sexual pain (hEDS females vs. males OR 4.13, CI 1.51–10.20, *p* = 0.003), and orgasm difficulty (hEDS females vs. males OR 4.70, CI 1.44–15.08, *p* = 0.006) (Table 4, Supplementary Table S4). In contrast, no significant differences were observed in females with HSD and males with HSD (Table 4). Thus, females with hEDS reported more sexual problems than males with hEDS.

### 3.5 Sex differences in genitourinary symptoms/comorbidities in patients with HSD and hEDS

To investigate whether the increased sexual problems reported by female patients with hEDS related to genitourinary symptoms, in a separate study, we examined whether sex differences existed in self-reported genitourinary symptoms/comorbidities in patients diagnosed with HSD or hEDS. We found that females diagnosed with hEDS reported more frequent urination (OR 3.9, CI 1.40–10.72, *p* = 0.008), dyspareunia (pain during sexual intercourse) (OR ∞, CI 4.04–∞, *p* < 0.0001), recurrent urinary tract infections (OR 8.27, CI 2.20–35.89, *p* = 0.0007), incontinence (urine leakage) (OR 11.57, CI 2.09–121.00, *p* = 0.002), pelvic floor dysfunction (OR 10.55, CI 1.91–110.30, *p* = 0.003), and recurrent yeast infections (OR ∞, CI 2.52–∞, *p* = 0.001) compared with males diagnosed with hEDS (Table 5, Supplementary Table S4). In contrast, fewer sex differences were observed in patients with HSD. Females diagnosed with HSD reported more frequent dyspareunia (pain during sexual intercourse) (OR 4.66, CI 1.54–14.06, *p* = 0.001), recurrent urinary tract infections (OR 6.21, CI 1.71–22.50, *p* = 0.0004), and recurrent yeast infections (OR 18.95, CI 1.16–309.80, *p* = 0.0005) compared with males diagnosed with hEDS (Table 5, Supplementary Table S4). Thus, females with hEDS experienced more genitourinary symptoms/comorbidities compared with females or males with HSD of either diagnosis.

**Table 5:** Sex differences in genitourinary symptoms/comorbidities in females and males with hEDS or HSD (*n* = 1,216).

Genitourinary symptoms	Female HSD^a^ (*n* = 937)	Male HSD^a^ (*n* = 39)	*p*-value^b^	Female hEDS^a^ (*n* = 210)	Male hEDS^a^ (*n* = 30)	*p*-value^a^
Frequent urination	372 (39.7)	15 (38.5)	0.99	79 (37.6)	4 (13.3)	**0** **.** **008** ^c^
Dyspareunia (pain during sexual intercourse)	289 (30.8)	3 (7.7)	**0** **.** **001**	67 (31.9)	0 (0.0)	**<0** **.** **0001**
Recurrent urinary tract infections	274 (29.2)	2 (5.1)	**0** **.** **0004**	78 (37.1)	2 (6.7)	**0** **.** **0007**
Incontinence (urine leakage)	248 (26.5)	5 (13.2)	0.09	63 (30.0)	1 (3.6)	**0** **.** **002**
Pelvic floor dysfunction	184 (19.6)	3 (7.7)	0.06	56 (26.7)	1 (3.3)	**0** **.** **003**
Recurrent yeast infections	181 (19.3)	0 (0.0)	**0** **.** **0005**	48 (22.9)	0 (0.0)	**0** **.** **001**
Recurrent vaginal bacterial infections	90 (9.6)	0 (0.0)	n/a	25 (11.9)	0 (0.0)	n/a
Pelvic floor spasms	122 (13.0)	2 (5.1)	0.22	35 (16.7)	3 (10.0)	0.43
Interstitial cystitis (inflammation of the bladder)	92 (9.8)	1 (2.6)	0.17	25 (11.9)	0 (0.0)	0.05
Bladder prolapse	59 (6.3)	0 (0.0)	0.16	85 (4.9)	0 (0.0)	0.09
Uterine prolapse	47 (5.0)	0 (0.0)	n/a	21 (10.0)	0 (0.0)	n/a
Rectal prolapse	55 (5.0)	2 (5.1)	0.99	22 (10.5)	2 (6.7)	0.75
Other	137 (14.6)	5 (12.8)	0.99	34 (16.2)	1 (3.3)	0.09
Unknown	72 (7.7)	3 (7.7)	0.99	17 (8.1)	6 (20.0)	0.05
No issues	150 (16.0)	24 (61.5)	**<0** **.** **0001**	28 (13.3)	20 (66.7)	**<0** **.** **0001**

^a^ Data shown as *n* (%).

^b^ *p*-values obtained using Fisher's exact test.

^c^ Bold indicates significant value.

## 4 Discussion

The objective of this exploratory study was to examine whether sex differences existed in sexual problems in patients diagnosed with hEDS or HSD compared with unhealthy, non-hypermobile chronic pain controls. We found that females with a diagnosis of hEDS self-reported more sexual issues, including sexual problems (69.1%), problems with sexual interest (41.4%), sexual pain (45.2%), and orgasm difficulty (34.3%) compared with EDS Clinic control females who had chronic pain but were not diagnosed with HSD or hEDS or any other form of EDS, including L-HSD and H-HSD. In contrast, males diagnosed with hEDS did not report sexual problems above those of EDS Clinic male chronic pain controls. These findings contrasted with patients diagnosed with HSD, where both males and females reported only one issue—more problems with sexual interest—compared with EDS Clinic controls. Survey studies have found that 12% of women in the general population experience sexual dysfunction related to desire, arousal, and/or orgasm (29, 30) and 13%–21% of women experience painful sex (31, 32). Our findings indicate a far higher percentage of sexual problems in females with hEDS than has been reported for the general population, similar to previous reports (17, 23–25). Furthermore, our findings may even be an underestimation of the prevalence of sexual problems in patients with hEDS because the controls in this study were older than patients with hEDS and sexual issues are known to increase with age. In addition, because the controls were patients with chronic pain rather than healthy controls, their sexual function may already have been impaired, thereby diluting differences between groups. We do not know what the primary diagnosis of the non-hypermobile control patients should be, and it is possible that they had fibromyalgia or another connective tissue condition such as rheumatoid arthritis. Thus, overall, we found that females with hEDS reported more sexual problems. Sexual problems were not reported more often in male patients with hEDS or female or male patients with HSD above chronic pain controls.

There are several key differences between this study and previous publications. This study is unique because we examine sex differences in sexual problems in a relatively large number of patients, which is necessary in order to have sufficient males. This study is also unique because we examine sexual problems in patients with physician-confirmed diagnoses of HSD and hEDS rather than relying on self-reported data of a physician diagnosis (23, 24). We found sex differences in many of the eight sexual problem screening questions that we examined in this study in patients with hEDS but few sexual problems in patients with HSD regardless of sex. In addition, this study is the first to include non-hypermobile controls with chronic pain who did not receive a diagnosis of hEDS or HSD but attended the EDS Clinic and took the same questionnaire. In future studies, we would also like to include healthy non-hypermobile controls. We expect differences in sexual problems between patients with hEDS and HSD vs. non-hypermobile healthy controls to be even greater than those in non-hypermobile controls with chronic pain.

Interestingly, when we examined genitourinary symptoms/comorbidities to determine whether these issues might contribute to our findings, we found sex differences in 30% (3/10) of genitourinary issues in patients with HSD vs. 60% (6/10) of patients with hEDS, with females reporting more issues than males (Table 5). The low number of males examined in this study precludes drawing clear conclusions, and future studies with more males are needed. Regardless, these data suggest that genitourinary symptoms/comorbidities in patients with hEDS vs. those with HSD may contribute, at least in part, to sexual problems. The current diagnostic criteria for hEDS select patients with collagen dysfunction by choosing individuals with stretchy skin, heel papules, etc. (3). We previously reported that patients with hEDS had more problems such as prolapses, hernias, and dislocations than those with HSD (10), suggesting that patients with hEDS may have a weaker collagen/extracellular matrix (ECM). The central role of collagen/ECM for maintaining the integrity of the pelvic region further suggests that injury to this area by physical trauma (sexual abuse, accidents, etc.), unstable hips, spine, knees, and pregnancy and/or infections may weaken or alter the ECM in this region, contributing to sexual problems. Additional factors that may promote ECM remodeling include neurological dysfunction (i.e., autonomic dysfunction), mast cell activation, and/or psychological issues (i.e., stress, post-traumatic stress disorder/PTSD, and abuse).

Sex differences are known to occur in sexual problems, with more widespread symptoms/issues reported by females than by males (31, 33). In a study of over 3,000 men and women from the US National Health and Social Life Survey, it was found that the greatest risk for sexual dysfunction (i.e., low desire, arousal dysfunction, and sexual pain) in women were urinary tract symptoms, poor health, stress, and abuse as a child (31). Men had the same risk factors for sexual dysfunction as women, with additional factors such as daily use of alcohol and sexual experience (i.e., ever had a male partner, think about sex <1×/week). Another sex difference observed in that study was that if the individual had low physical or emotional satisfaction and/or low general happiness, women were at increased risk of developing low desire, arousal dysfunction, and sexual pain (i.e., widespread issues), while males had an increased risk only for erectile dysfunction, which was confirmed in a later study (31, 33). An important finding from the McCabe et al. study was that most men receive treatment for erectile dysfunction with their primary care physician, while concerns of most women go unaddressed (33). This situation may be further complicated in women with hEDS because of symptoms related to hypermobility, such as recurrent hip subluxation and increased rates of uterine, bladder, and rectal prolapse, compared with other women.

### 4.1 Limitations

There are several limitations to this study. The retrospective design of the study precludes establishing causal relationships. The site of the study is a tertiary care center and findings from this study may not represent other regions of the US or the world. In addition, symptoms/comorbidities were self-reported and not validated through another method. Importantly, the small number of males in this study limits the conclusions that can be drawn regarding male sexual problems. However, given the very limited availability of data in the literature on males with hEDS and HSD, information provided in this study is important. This study should be repeated in the future with a higher number of males. Another possible limitation is that we do not have similar numbers of patients per group, although this distribution represents the total number of patients seen at our EDS Clinic who took the questions about sexual problems. This study was an exploratory evaluation of sexual problems in female and male patients with hEDS and HSD. For this reason, we did not adjust for multiple comparisons for symptoms, and therefore, there is a risk of type I errors (false positives). Findings from this study will need to be confirmed with future research using validated questionnaires such as the FSFI for women and the International Index of Erectile Function (IIEF) for men. Future studies are also needed to examine whether the key findings of this study can be verified when medical records are examined. The strengths of this study are that patients with hEDS/HSD were diagnosed using the most recent 2017 diagnostic and other criteria (2–4) by physician experts, and we used sexual problem screening questions that have been previously validated (28). An additional strength is the large study population that contained an internal control group that was not diagnosed with hEDS or HSD (i.e., not hypermobile) but underwent the same diagnostic process and answered the same sexual problem questions.

### 4.2 Clinical implications

Our findings indicate that sexual problems exist, especially in patients with hEDS. Currently, all patients who attend our EDS Clinic receive referrals to physical and occupational therapy. Our findings suggest that patients with relevant symptoms should be considered for evaluation/referral to gynecology, urology, and pelvic floor physical therapy to assess their genitourinary and sexual problems.

## 5 Conclusion

Our study of males and females with physician-verified hEDS and HSD found that females with hEDS reported more widespread sexual problems than chronic pain controls, males, or patients with HSD. Females with hEDS also reported more genitourinary symptoms/comorbidities than females with HSD. More research is needed on sexual problems in males with hEDS and HSD using larger numbers.

## Data Availability

The raw data supporting the conclusions of this article will be made available by the authors, without undue reservation.
