# Supplementary material for: Females with hypermobile Ehlers–Danlos syndrome self-report more sexual problems than chronic pain controls without hypermobility, males, or patients with hypermobile spectrum disorders
Source: Front Reprod Health. 2026 Apr 1;8:1753684. doi: 10.3389/frph.2026.1753684 (PMC13079633; doi:10.3389/frph.2026.1753684)
Supplement: Supplementary file 1 [file Datasheet1.pdf]

Supplemental Data

Females with hypermobile Ehlers-Danlos syndrome self-report more sexual problems than chronic pain controls without hypermobility, males or hypermobile spectrum disorders patients

Cynthia E. Neville, PT, DPT;[1]* Frances C. Wilson, BS;[2]* DeLisa Fairweather, PhD;[2,4,5]*†# Melissa K. Caywood, PT, DPT;[1] Katherine Gegoutchadze, BS, BA;[3] Lincoln E. Rozen;[2,3] Nick A. Farahani;[3] Chrisandra L. Shufelt, MD;[4] Dacre R.T. Knight, MD, MS;[4]† Shilpa N. Gajarawala, DMSc, MPAS, PA-C;[4]† Katelyn A. Bruno, PhD[3]†#

[1]Department of Physical Medicine and Rehabilitation, Mayo Clinic, 4500 San Pablo Road, Jacksonville, Florida, USA
[2]Department of Cardiovascular Medicine, Mayo Clinic, 4500 San Pablo Road, Jacksonville, Florida, USA
[3]Division of Cardiovascular Medicine, University of Florida, 1329 SW 16th Street, Gainesville, Florida, USA
[4]Department of General Internal Medicine, Mayo Clinic, 4500 San Pablo Road, Jacksonville, Florida, USA
[5]Center for Clinical and Translational Science, Mayo Clinic, 200 First Street SW, Rochester, Minnesota, USA

*Equal contribution and co-first authors: CEN, FCW, DF contributed equally to this work and share first authorship.

†Equal contribution and senior authorship: DF, DRTK, SNG, KAB contributed equally to this work and share senior authorship.

#Co-corresponding author: DeLisa Fairweather, PhD, FAHA, FHFSA; Professor, Mayo Clinic, Department of Cardiovascular Medicine, 4500 San Pablo Road, Jacksonville, FL 32224, USA; Email: Fairweather.DeLisa@mayo.edu; ORCID: 0000-0003-3093-1810.

#Co-corresponding author: Katelyn A. Bruno, PhD, FHFSA, FACC, Assistant Professor, University of Florida, Department of Medicine, Division of Cardiovascular Medicine, 1329 SW 16th Street, Gainesville, Florida 32610-0288, USA, Katelyn.Bruno@medicine.ufl.edu; ORCID: 0000-0001-8980-6059.

Supplemental Tables

Supplementary Table 1. Patient demographics for females (*n* = 1,312)

	**Chronic pain control (*n* = 165) *n* (%)**	**HSD (*n* = 937) *n* (%)**	**hEDS (*n* = 210) *n* (%)**	***P* value[a]**
**Age**				
mean (range)	40.6 (18.0-76.5)	35.3 (18.0-70.9)****	35.6 (18.2-71.3)###	**<0.0001[b]**
**Race**				
American Indian/ Alaska Native	2 (1.2)	14 (1.5)	6 (2.9)	0.99
Asian	2 (1.2)	20 (2.1)	1 (0.5)	0.99
Black/ African American	3 (1.8)	20 (2.1)	11 (5.2)	0.43
Native Hawaii/ Pacific Islander	0 (0.0)	1 (0.1)	0 (0.0)	0.99
White	157 (95.2)	888 (94.8)	200 (95.2)	0.98
Other	0 (0.0)	27 (2.9)	5 (2.4)	0.44
Unknown	3 (1.8)	7 (0.7)	3 (1.4)	0.99
**Ethnicity**				
Hispanic/Latino	8 (4.8)	77 (8.2)	11 (5.2)	0.68
Not Hispanic/Latino	147 (89.1)	839 (89.5)	196 (93.3)	0.22
Not disclosed	10 (6.1)	21 (2.2)*	3 (1.4)#	0.19
**Highest level of education**				
Some high school	2 (1.2)	20 (2.1)	3 (1.4%)	0.99
High school graduate	16 (9.7)	74 (7.9)	11 (5.2%)	0.24
Some college	44 (26.7)	202 (21.6)	51 (24.3%)	0.25
Trade/ technical/ vocational school				0.99
Associate's degree	4 (2.4)	47 (5.0)	7 (3.3)	0.25
Bachelor's degree	13 (7.9)	110 (11.7)	28 (13.3)	0.62
Master's degree	28 (17.0)	138 (14.7)	37 (17.6)	0.44
Professional/ doctorate degree	10 (6.1)	59 (6.3)	16 (7.6)	0.99
**History of smoking**				
Current smoker	13 (7.9)	80 (8.5)	18 (8.6)	0.97
Past smoker	19 (11.5)	161 (17.2)	42 (20.0)#	0.08
No history	132 (80.0)	687 (73.3)	149 (71.0)	0.11
Unknown	1 (0.6)	9 (1.0)	1 (0.5)	0.99

**History of secondhand smoke exposure**				
Secondhand smoke history	73 (44.2)	438 (46.7)	87 (41.4)	0.35
No history	92 (55.8)	485 (51.8)	122 (58.1)	0.20
Unknown	0 (0.0)	14 (1.5)	1 (0.5)	0.99
**History of alcohol consumption**				
Current alcohol consumption	87 (52.7)	509 (54.3)	130 (61.9)*	0.10
Past alcohol consumption	38 (23.0)	246 (26.3)	54 (25.7)	0.71
No history	39 (23.6)	178 (19.0)	24 (11.4)##,^^	**0.005**
Unknown	1 (0.6)	4 (0.4)	2 (1.0)	0.99

[a] P values for continuous variables were calculated using a Student's *t* test for parametric data and a Mann-Whitney test for non-parametric data. A Fisher's exact test was used to determine p values for categorical variables. [b]Bold indicates significant value. *compares control to HSD, #compares control to hEDS, ^compares HSD to hEDS; *,#,^ $p<0.05$; **,##,^^ $p<0.01$, ***,###,^^^ $p<0.001$, ****,####,^^^^ $p<0.0001$.

Supplementary Table 2. Patient demographics for males (n = 95)

	**Chronic pain control (*n* = 26) *n* (%)**	**HSD (*n* = 39) *n* (%)**	**hEDS (*n* = 30) *n* (%)**	***P* value[a]**
**Age**				
Mean (range)	37.9 (18.6-68.6)	33.3 (18.5-81.9)	28.1 (18.0-52.0)#	**0.018**
**Race**				
American Indian/ Alaska Native	0 (0.0)	0 (0.0)	1 (3.3)	0.59
Asian	1 (3.8)	0 (0.0)	0 (0.0)	0.27
Black/ African American	0 (0.0)	1 (2.6)	2 (6.7)	0.48
Native Hawaii/ Pacific Islander	0 (0.0)	0 (0.0)	0 (0.0)	0.99
White	23 (88.5)	38 (97.4)	29 (96.7)	0.37
Other	0 (0.0)	0 (0.0)	1 (3.3)	0.59
Unknown	2 (7.7)	0 (0.0)	0 (0.0)	0.07
**Ethnicity**				
Hispanic/Latino	0 (0.0)	2 (5.1)	1 (3.3)	0.51
Not Hispanic/Latino	21 (80.1)	36 (92.3)	29 (96.7)	0.11
Not disclosed	5 (19.2)	1 (2.6)*	0 (0.0)#	**0.006**
**Highest level of education**				
Some high school	0 (0.0%)	2 (5.1)	0 (0.0)	0.23
High school graduate	2 (7.7%)	5 (12.8)	5 (16.7)	0.60
Some college	7 (26.9%)	12 (30.8)	8 (26.7)	0.91
Trade/ Technical/ Vocational school	1 (3.8%)	1 (2.6)	0 (0.0)	0.59
Associate's degree	3 (11.5%)	3 (7.7)	5 (16.7)	0.51
Bachelor's degree	8 (30.8%)	8 (20.5)	6 (20.0)	0.56
Master's degree	3 (11.5%)	4 (10.3)	3 (10.0)	0.98
Professional/ doctorate degree	2 (7.7%)	4 (10.3)	3 (10.0)	0.95
**History of smoking**				
Current smoker	2 (7.7)	6 (15.4)	6 (20.0)	0.43
Past smoker	5 (19.2)	4 (10.3)	3 (10.0)	0.49
No history	19 (73.1)	29 (74.4)	21 (70.0)	0.92
Unknown	0 (0.0)	0 (0.0)	0 (0.0)	-
**History of secondhand smoke exposure**				
Secondhand smoke exposure	11 (42.3)	16 (41.0)	9 (30.0)	0.56

No history	15 (57.7)	23 (59.0)	21 (70.0)	0.56
Unknown	0 (0.0)	0 (0.0)	0 (0.0)	-
**History of alcohol consumption**				
Current alcohol consumption	14 (53.8)	18 (46.1)	13 (43.3)	0.72
History of alcohol consumption	8 (30.8)	10 (2.56)	10 (33.3)	0.77
No history	4 (15.4)	11 (28.2)	7 (23.3)	0.49
Unknown	0 (0.0)	0 (0.0)	0 (0.0)	-

[a] P values for continuous variables were calculated using a Student's *t* test for parametric data and a Mann-Whitney test for non-parametric data. A Fisher's exact test was used to determine p values for categorical variables. [b]Bold indicates significant value. *compares control to HSD, #compares control to hEDS, ^compares HSD to hEDS; *,#,^ $p<0.05$; **,##,^^ $p<0.01$, ***,###,^^^ $p<0.001$, ****,####,^^^^ $p<0.0001$.

Supplementary Table 3. Odds ratios with 95% confidence intervals for sexual problems in males and females with hEDS or HSD vs. chronic pain controls

	**Female OR (95% CI)**[a]	***P* value**[b]	**Male OR (95% CI)**	***P* value**
Chronic pain control vs. HSD				
Sexual interest problems	1.55 (1.09 - 2.21)	**0.019**[c]	6.00 (1.21 - 28.29)	**0.018**
Chronic pain control vs. hEDS				
Sexual problems	1.69 (1.11 - 2.58)	**0.018**	1.22 (0.43 - 3.62)	0.79
Sexual interest problems	1.67 (1.09 - 2.56)	**0.023**	4.36 (0.84 - 21.65)	0.09
Sexual pain	1.85 (1.21 - 2.80)	**0.006**	0.67 (0.19 - 2.32)	0.74
Orgasm difficulty	1.74 (1.11 - 2.77)	**0.022**	0.85 (0.19 - 3.94)	0.99

[a] Data shown as odds ratio (OR) and 95% confidence intervals (CI). [b] P values obtained using Fisher's exact test. [c] Bold indicates significant value.

Supplementary Table 4. Odds ratios with 95% confidence intervals for sex differences in sexual and genitourinary problems in hEDS and HSD patients

	**HSD OR (95% CI)**[a]	***P* value**[b]	**hEDS OR (95% CI)**	***P* value**
Female vs. Male				
Sexual problems	1.46 (0.78 - 2.82)	0.24	2.92 (1.33 - 6.25)	**0.007**[c]
Sexual pain	1.38 (0.70 - 2.66)	0.43	4.13 (1.51 - 10.20)	**0.003**
Orgasm difficulty	1.66 (0.74 - 3.71)	0.21	4.70 (1.44 - 15.08)	**0.006**
Female vs. Male				
Frequent urination	1.05 (0.55 - 1.98)	0.99	3.9 (1.40 - 10.72)	**0.008**
Dyspareunia (pain during sexual intercourse)	4.66 (1.54 - 14.06)	**0.001**	∞ (4.04 - ∞)	**<0.0001**
Recurrent urinary tract infections	6.21 (1.71 - 22.50)	**0.0004**	8.27 (2.20 - 35.89)	**0.0007**
Incontinence (urine leakage)	2.20 (0.88 - 5.47)	0.09	11.57 (2.09 - 121.00)	**0.002**
Pelvic floor dysfunction	2.55 (0.84 - 7.74)	0.06	10.55 (1.91 - 110.30)	**0.003**
Recurrent yeast infections	18.95 (1.16 - 309.80)	**0.0005**	∞ (2.52 - ∞)	**0.001**

[a] Data shown as odds ratio (OR) and 95% confidence intervals (CI). [b] P values obtained using Fisher's exact test. [c] Bold indicates significant value.
