# Supplementary material for: Females with hypermobile Ehlers–Danlos syndrome self-report more sexual problems than chronic pain controls without hypermobility, males, or patients with hypermobile spectrum disorders
Source: Front Reprod Health. 2026 Apr 1;8:1753684. doi: 10.3389/frph.2026.1753684 (PMC13079633; doi:10.3389/frph.2026.1753684)
Supplement: Supplementary file 2 [file Table1.docx]

Supplemental Data

**Females with hypermobile Ehlers-Danlos syndrome self-report more sexual problems than chronic pain controls without hypermobility, males or hypermobile spectrum disorders patients**

Cynthia E. Neville, PT, DPT;^1^* Frances C. Wilson, BS;^2^* DeLisa Fairweather, PhD;^2,4,5^*†# Melissa K. Caywood, PT, DPT;^1^ Katherine Gegoutchadze, BS, BA;^3^ Lincoln E. Rozen;^2,3^ Nick A. Farahani;^3^ Chrisandra L. Shufelt, MD;^4^ Dacre R.T. Knight, MD, MS;^4^† Shilpa N. Gajarawala, DMSc, MPAS, PA-C;^4^† Katelyn A. Bruno, PhD^3^†#

^1^Department of Physical Medicine and Rehabilitation, Mayo Clinic, 4500 San Pablo Road, Jacksonville, Florida, USA

^2^Department of Cardiovascular Medicine, Mayo Clinic, 4500 San Pablo Road, Jacksonville, Florida, USA

^3^Division of Cardiovascular Medicine, University of Florida, 1329 SW 16th Street, Gainesville, Florida, USA

^4^Department of General Internal Medicine, Mayo Clinic, 4500 San Pablo Road, Jacksonville, Florida, USA

^5^Center for Clinical and Translational Science, Mayo Clinic, 200 First Street SW, Rochester, Minnesota, USA

*Equal contribution and co-first authors: CEN, FCW, DF contributed equally to this work and share first authorship.

†Equal contribution and senior authorship: DF, DRTK, SNG, KAB contributed equally to this work and share senior authorship.

#Co-corresponding author: DeLisa Fairweather, PhD, FAHA, FHFSA; Professor, Mayo Clinic, Department of Cardiovascular Medicine, 4500 San Pablo Road, Jacksonville, FL 32224, USA; Email: Fairweather.DeLisa@mayo.edu; ORCID: 0000-0003-3093-1810.

#Co-corresponding author: Katelyn A. Bruno, PhD, FHFSA, FACC, Assistant Professor, University of Florida, Department of Medicine, Division of Cardiovascular Medicine, 1329 SW 16th Street, Gainesville, Florida 32610-0288, USA, Katelyn.Bruno@medicine.ufl.edu; ORCID: 0000-0001-8980-6059.

**Supplemental Tables**

Supplementary Table 1. Patient demographics for females (*n* = 1,312)

|  | **Chronic pain control**  **(*n* = 165) *n* (%)** | **HSD**  **(*n* = 937) *n* (%)** | **hEDS**  **(*n* = 210) *n* (%)** | ***P* value*^a^*** |
| --- | --- | --- | --- | --- |
| **Age** |  |  |  |  |
| mean (range) | 40.6  (18.0-76.5) | 35.3  (18.0-70.9)**** | 35.6  (18.2-71.3)### | **<0.0001***^b^* |
| **Race** |  |  |  |  |
| American Indian/ Alaska Native | 2 (1.2) | 14 (1.5) | 6 (2.9) | 0.99 |
| Asian | 2 (1.2) | 20 (2.1) | 1 (0.5) | 0.99 |
| Black/ African American | 3 (1.8) | 20 (2.1) | 11 (5.2) | 0.43 |
| Native Hawaii/ Pacific Islander  White  Other  Unknown | 0 (0.0)  157 (95.2)  0 (0.0)  3 (1.8) | 1 (0.1)  888 (94.8)  27 (2.9)  7 (0.7) | 0 (0.0)  200 (95.2)  5 (2.4)  3 (1.4) | 0.99  0.98  0.44  0.99 |
| **Ethnicity** |  |  |  |  |
| Hispanic/Latino | 8 (4.8) | 77 (8.2) | 11 (5.2) | 0.68 |
| Not Hispanic/Latino | 147 (89.1) | 839 (89.5) | 196 (93.3) | 0.22 |
| Not disclosed | 10 (6.1) | 21 (2.2)* | 3 (1.4)# | 0.19 |
| **Highest level of education** |  |  |  |  |
| Some high school | 2 (1.2) | 20 (2.1) | 3 (1.4%) | 0.99 |
| High school graduate | 16 (9.7) | 74 (7.9) | 11 (5.2%) | 0.24 |
| Some college  Trade/ technical/ vocational school | 44 (26.7) | 202 (21.6) | 51 (24.3%) | 0.25  0.99 |
| Associate’s degree | 4 (2.4) | 47 (5.0) | 7 (3.3) | 0.25 |
| Bachelor’s degree | 13 (7.9) | 110 (11.7) | 28 (13.3) | 0.62 |
| Master’s degree | 28 (17.0) | 138 (14.7) | 37 (17.6) | 0.44 |
| Professional/ doctorate degree | 10 (6.1) | 59 (6.3) | 16 (7.6) | 0.99 |
| **History of smoking** |  |  |  |  |
| Current smoker | 13 (7.9) | 80 (8.5) | 18 (8.6) | 0.97 |
| Past smoker | 19 (11.5) | 161 (17.2) | 42 (20.0)# | 0.08 |
| No history | 132 (80.0) | 687 (73.3) | 149 (71.0) | 0.11 |
| Unknown | 1 (0.6) | 9 (1.0) | 1 (0.5) | 0.99 |
| **History of secondhand smoke exposure** |  |  |  |  |
| Secondhand smoke history | 73 (44.2) | 438 (46.7) | 87 (41.4) | 0.35 |
| No history | 92 (55.8) | 485 (51.8) | 122 (58.1) | 0.20 |
| Unknown | 0 (0.0) | 14 (1.5) | 1 (0.5) | 0.99 |
| **History of alcohol consumption** |  |  |  |  |
| Current alcohol consumption | 87 (52.7) | 509 (54.3) | 130 (61.9)* | 0.10 |
| Past alcohol consumption | 38 (23.0) | 246 (26.3) | 54 (25.7) | 0.71 |
| No history | 39 (23.6) | 178 (19.0) | 24 (11.4)##,^^ | **0.005** |
| Unknown | 1 (0.6) | 4 (0.4) | 2 (1.0) | 0.99 |

*^a^* P values for continuous variables were calculated using a Student’s *t* test for parametric data and a Mann-Whitney test for non-parametric data. A Fisher’s exact test was used to determine p values for categorical variables. *^b^*Bold indicates significant value. *compares control to HSD, #compares control to hEDS, ^compares HSD to hEDS; *,#,^ *p*<0.05; **,##,^^ *p*<0.01, ***,###,^^^ *p*<0.001, ****,####,^^^^ *p*<0.0001.

Supplementary Table 2. Patient demographics for males (*n* = 95)

|  | **Chronic pain control**  **(*n* = 26) *n* (%)** | **HSD**  **(*n* = 39) *n* (%)** | **hEDS**  **(*n* = 30) *n* (%)** | ***P* value*^a^*** |
| --- | --- | --- | --- | --- |
| **Age** |  |  |  |  |
| Mean (range) | 37.9 (18.6-68.6) | 33.3 (18.5-81.9) | 28.1 (18.0-52.0)# | **0.018** |
| **Race** |  |  |  |  |
| American Indian/ Alaska Native | 0 (0.0) | 0 (0.0) | 1 (3.3) | 0.59 |
| Asian | 1 (3.8) | 0 (0.0) | 0 (0.0) | 0.27 |
| Black/ African American | 0 (0.0) | 1 (2.6) | 2 (6.7) | 0.48 |
| Native Hawaii/ Pacific Islander  White  Other  Unknown | 0 (0.0)  23 (88.5)  0 (0.0)  2 (7.7) | 0 (0.0)  38 (97.4)  0 (0.0)  0 (0.0) | 0 (0.0)  29 (96.7)  1 (3.3)  0 (0.0) | 0.99  0.37  0.59  0.07 |
| **Ethnicity** |  |  |  |  |
| Hispanic/Latino | 0 (0.0) | 2 (5.1) | 1 (3.3) | 0.51 |
| Not Hispanic/Latino | 21 (80.1) | 36 (92.3) | 29 (96.7) | 0.11 |
| Not disclosed | 5 (19.2) | 1 (2.6)* | 0 (0.0)# | **0.006** |
| **Highest level of education** |  |  |  |  |
| Some high school | 0 (0.0%) | 2 (5.1) | 0 (0.0) | 0.23 |
| High school graduate | 2 (7.7%) | 5 (12.8) | 5 (16.7) | 0.60 |
| Some college  Trade/ Technical/ Vocational school | 7 (26.9%)  1 (3.8%) | 12 (30.8)  1 (2.6) | 8 (26.7)  0 (0.0) | 0.91  0.59 |
| Associate’s degree | 3 (11.5%) | 3 (7.7) | 5 (16.7) | 0.51 |
| Bachelor’s degree | 8 (30.8%) | 8 (20.5) | 6 (20.0) | 0.56 |
| Master’s degree | 3 (11.5%) | 4 (10.3) | 3 (10.0) | 0.98 |
| Professional/ doctorate degree | 2 (7.7%) | 4 (10.3) | 3 (10.0) | 0.95 |
| **History of smoking** |  |  |  |  |
| Current smoker | 2 (7.7) | 6 (15.4) | 6 (20.0) | 0.43 |
| Past smoker | 5 (19.2) | 4 (10.3) | 3 (10.0) | 0.49 |
| No history | 19 (73.1) | 29 (74.4) | 21 (70.0) | 0.92 |
| Unknown | 0 (0.0) | 0 (0.0) | 0 (0.0) | - |
| **History of secondhand smoke exposure** |  |  |  |  |
| Secondhand smoke exposure | 11 (42.3) | 16 (41.0) | 9 (30.0) | 0.56 |
| No history | 15 (57.7) | 23 (59.0) | 21 (70.0) | 0.56 |
| Unknown | 0 (0.0) | 0 (0.0) | 0 (0.0) | - |
| **History of alcohol consumption** |  |  |  |  |
| Current alcohol consumption | 14 (53.8) | 18 (46.1) | 13 (43.3) | 0.72 |
| History of alcohol consumption | 8 (30.8) | 10 (2.56) | 10 (33.3) | 0.77 |
| No history | 4 (15.4) | 11 (28.2) | 7 (23.3) | 0.49 |
| Unknown | 0 (0.0) | 0 (0.0) | 0 (0.0) | - |

*^a^* P values for continuous variables were calculated using a Student’s *t* test for parametric data and a Mann-Whitney test for non-parametric data. A Fisher’s exact test was used to determine p values for categorical variables. *^b^*Bold indicates significant value. *compares control to HSD, #compares control to hEDS, ^compares HSD to hEDS; *,#,^ *p*<0.05; **,##,^^ *p*<0.01, ***,###,^^^ *p*<0.001, ****,####,^^^^ *p*<0.0001.

Supplementary Table 3. Odds ratios with 95% confidence intervals for sexual problems in males and females with hEDS or HSD vs. chronic pain controls

|  | Female  OR (95% CI)*^a^* | *P* value*^b^* | Male  OR (95% CI) | *P* value |
| --- | --- | --- | --- | --- |
| *Chronic pain control vs. HSD* | | | | |
| Sexual interest problems | 1.55 (1.09 - 2.21) | **0.019***^c^* | 6.00 (1.21 - 28.29) | **0.018** |
| *Chronic pain control vs. hEDS* | | | | |
| Sexual problems | 1.69 (1.11 - 2.58) | **0.018** | 1.22 (0.43 - 3.62) | 0.79 |
| Sexual interest problems | 1.67 (1.09 - 2.56) | **0.023** | 4.36 (0.84 - 21.65) | 0.09 |
| Sexual pain | 1.85 (1.21 - 2.80) | **0.006** | 0.67 (0.19 - 2.32) | 0.74 |
| Orgasm difficulty | 1.74 (1.11 - 2.77) | **0.022** | 0.85 (0.19 - 3.94) | 0.99 |

*^a^* Data shown as odds ratio (OR) and 95% confidence intervals (CI). *^b^* P values obtained using Fisher's exact test. *^c^* Bold indicates significant value.

Supplementary Table 4. Odds ratios with 95% confidence intervals for sex differences in sexual and genitourinary problems in hEDS and HSD patients

|  | **HSD**  **OR (95% CI)***^a^* | ***P* value***^b^* | **hEDS**  **OR (95% CI)** | ***P* value** |
| --- | --- | --- | --- | --- |
| ***Female vs. Male*** |  |  |  |  |
| Sexual problems | 1.46 (0.78 - 2.82) | 0.24 | 2.92 (1.33 - 6.25) | **0.007***^c^* |
| Sexual pain | 1.38 (0.70 - 2.66) | 0.43 | 4.13 (1.51 - 10.20) | **0.003** |
| Orgasm difficulty | 1.66 (0.74 - 3.71) | 0.21 | 4.70 (1.44 - 15.08) | **0.006** |
| ***Female vs. Male*** |  |  |  |  |
| Frequent urination | 1.05 (0.55 - 1.98) | 0.99 | 3.9 (1.40 - 10.72) | **0.008** |
| Dyspareunia (pain during sexual intercourse) | 4.66 (1.54 - 14.06) | **0.001** | ∞ (4.04 - ∞) | **<0.0001** |
| Recurrent urinary tract infections | 6.21 (1.71 - 22.50) | **0.0004** | 8.27 (2.20 - 35.89) | **0.0007** |
| Incontinence (urine leakage) | 2.20 (0.88 - 5.47) | 0.09 | 11.57 (2.09 - 121.00) | **0.002** |
| Pelvic floor dysfunction | 2.55 (0.84 - 7.74) | 0.06 | 10.55 (1.91 - 110.30) | **0.003** |
| Recurrent yeast infections | 18.95 (1.16 - 309.80) | **0.0005** | ∞ (2.52 - ∞) | **0.001** |

*^a^* Data shown as odds ratio (OR) and 95% confidence intervals (CI). *^b^* P values obtained using Fisher's exact test. *^c^* Bold indicates significant value.
